# Expert-rated accuracy and safety of patient-facing dental radiology responses from three AI platform configurations: a comparative evaluation

**DOI:** 10.3389/froh.2026.1937905

**Published:** 2026-10-21

**Authors:** Deepika Kapoor, Deepanshu Garg, Ashutosh Nirola, M. K. Sunil, Pradhuman Verma, Santosh Kumar Tadakamadla

**Affiliations:** 1 Department of Pediatric and Preventive Dentistry, Luxmi Bai Institute of Dental Sciences and Hospital, Patiala, India; 2 Department of Oral Medicine and Oral Pathology, Griffith School of Medicine and Dentistry, Southport, QLD, Australia; 3 Department of Periodontics, Luxmi Bai Institute of Dental Sciences and Hospital, Patiala, India; 4 Department of Oral Medicine and Radiology, Dashmesh Institute of Research and Dental Sciences, Faridkot, India; 5 Department of Oral Medicine & Radiology, Dr Ziauddin Ahmad Dental College & Hospital, Aligarh Muslim University Campus, Aligarh, India; 6 Dentistry and Oral Health, La Trobe University, Bendigo, VIC, Australia

**Keywords:** artificial intelligence, consumer health information, dental radiology, large language models, misinformation, patient education, radiation safety

## Abstract

**Background:**

Consumer-facing artificial intelligence platforms are increasingly used to obtain health information, but the quality of their responses to dental radiology questions requires evaluation. This study compared expert ratings of single responses generated by three selected consumer-facing AI platform configurations to common patient-facing dental radiology questions.

**Methods:**

Twenty expert-reviewed English-language questions were submitted using one standardized prompt to the investigator-recorded, user-visible ChatGPT-5.5 configuration with Thinking enabled, Gemini 3 Flash free configuration, and Microsoft Copilot 2026 free configuration during late April 2026. One response was collected from each platform for each question, producing 60 responses. Three Oral and Maxillofacial Radiologists independently rated coded responses with platform labels concealed, assessing accuracy, relevance, clarity, completeness, safety, triage appropriateness and misinformation. The 20 questions were treated as the primary paired experimental units. Evaluator-averaged scores were compared across platform configurations using Friedman tests followed by question-level exact paired comparisons, while individual evaluator ratings were used descriptively and for agreement assessment.

**Results:**

The selected ChatGPT configuration received more favourable ratings than Gemini and Copilot for accuracy, relevance, safety and misinformation. It also received more favourable clarity and completeness ratings than Copilot. Gemini received more favourable question-level ratings than Copilot for clarity and completeness, but not for relevance. No significant differences were observed between ChatGPT and Gemini for clarity or completeness. Triage was applicable only to Question 20 and was reported descriptively. Considerable ceiling effects and tied ratings were observed, including 100% maximum ratings for ChatGPT accuracy and safety.

**Conclusion:**

Under the specified testing conditions, expert ratings differed across the single outputs generated by the selected platform configurations. The findings are limited by unequal platform configurations, one generation per question, unavailable historical interface metadata, a relatively homogeneous informational question set, ceiling effects, unverified masking, score–content inconsistencies and the absence of patient validation. They do not establish general model superiority, response repeatability, clinical readiness or patient benefit.

## 1 Introduction

The rapid advancement of artificial intelligence (AI), particularly deep learning and natural language processing, has influenced dental radiology by supporting image interpretation, diagnostic workflows, and clinical decision-making (1, 2). AI-driven systems have demonstrated potential applications in detecting dental caries, periodontal disease, periapical lesions, and other maxillofacial abnormalities (3, 4).

In parallel with these developments, large language models (LLMs), a class of generative artificial intelligence (AI) systems capable of producing human-like natural language responses, have emerged as widely accessible tools for delivering health-related information. Popular consumer-facing platforms include ChatGPT, Gemini, and Microsoft Copilot (5). Members of the public may consult these platforms when seeking explanations of health-related procedures, including dental radiographs and cone-beam computed tomography (6). Their ability to provide immediate, human-like responses has positioned them as potential instruments for patient education and decision support (7).

However, the use of LLMs in dental radiology raises important concerns. Patients commonly have questions about radiation exposure, imaging frequency, pregnancy, childhood imaging, and cancer risk. Inaccurate or incomplete AI-generated explanations could plausibly affect perceptions or decisions, but patient anxiety, imaging acceptance, healthcare-seeking behaviour, and clinical outcomes were not measured in this study (8).

Recent studies evaluating LLM performance in dentistry have primarily focused on diagnostic accuracy and clinical reasoning. Moura et al. reported variability in AI-assisted pulpal and periradicular diagnosis (9), while Azadi et al. compared chatbot responses to case-based oral and maxillofacial surgery questions (10). Other investigations have assessed oral mucosal lesion diagnosis and third-molar decision-making (7, 8). These studies indicate that performance depends on the task, platform, and evaluation method.

Beyond diagnostic contexts, emerging evidence indicates that LLM responses may also exhibit issues such as fabricated references, inconsistent reliability, and variability in response quality when addressing complex or controversial topics in oral medicine and radiology (11). Furthermore, studies evaluating AI for patient education in oral radiology have shown that although responses may be scientifically adequate, they often lack appropriate readability and patient-centred communication, limiting their practical applicability (12).

Previous patient-facing dental AI studies have evaluated dimensions beyond accuracy. Helvacioglu-Yigit et al. (12) assessed the quality and readability of AI-generated patient education in oral radiology. Kapoor et al. (13), with overlapping authors, compared ChatGPT, Gemini and Copilot responses to paediatric dentistry questions across accuracy, clarity, completeness, relevance and misleading information. The present study extends this work to patient-facing dental radiology and radiation-safety questions, incorporating an explicit safety domain, an item-specific evidence framework, transparent response and evaluator-rating data, and question-level paired analyses. Its contribution is therefore an incremental, task-specific evaluation rather than the first multidomain comparison of these platforms.

Access to reliable oral health information remains uneven, particularly in rural and underserved communities where barriers to professional care and lower health literacy may coexist (14–17). Consumer-facing AI tools may provide a supplementary source of general information, but their effect on patient understanding, anxiety, care-seeking, or acceptance of radiographic imaging has not been established.

Given the accessibility of consumer-facing generative AI platforms, it is important to examine the quality of patient-facing information generated under specific testing conditions. Therefore, this study aimed to compare expert ratings of single responses generated by three selected consumer-facing AI platform configurations to patient-centred dental radiology questions. The responses were evaluated for accuracy, relevance, clarity, completeness, safety, triage appropriateness, and misinformation. The study was not designed to establish the intrinsic or general superiority of any underlying model family.

## 2 Materials and methods

This study was designed as a comparative evaluation using coded responses with unverified evaluator masking of expert-rated informational quality and safety in responses generated by selected consumer-facing AI platform configurations to patient-centred dental radiology questions. It examined potential concerns associated with relying on these fixed informational outputs rather than clinical reliability or actual patient decision-making. Ethical approval was obtained from the Institutional Ethics Committee of Luxmi Bai Institute of Dental Sciences and Hospital, Patiala, India (Approval No. LBDH/26/377; dated 06 April 2026). The completed TRIPOD-LLM reporting checklist is provided in Supplementary File 4.

### 2.1 Question set development and expert review

A structured question set was developed using a multi-stage evidence-based process. Initially, a comprehensive review of international guidelines, radiation safety recommendations, professional association resources, and publicly available patient information sources was undertaken. Sources consulted included the Health Physics Society (HPS) Specialists in Radiation Protection (18), Health Physics Society Dental Radiation FAQs (19), Royal College of Dental Surgeons of Ontario (20), American Dental Association (ADA) patient information resources (21, 22), American Association of Orthodontists (23), International Atomic Energy Agency (IAEA) guidance on dental radiography, including specific guidance relating to pregnant women and children (24, 25), and the American Academy of Oral and Maxillofacial Radiology (AAOMR) (26).

To improve ecological relevance and ensure that the question set reflected common public information-seeking patterns, frequently searched online queries relating to dental radiation safety were identified using the web-based search analytics tool AlsoAsked (https://alsoasked.com) (27). The keyword “dental radiation safety” was entered on 10 April 2026 using a standardized search strategy across New York City (USA), Mumbai (India), Toronto, Ontario (Canada), and London (United Kingdom). Recurring questions, concerns, and misconceptions were reviewed during question development. An initial pool of 57 patient-centred questions was generated, covering general radiation safety, frequency and necessity of imaging, radiation risks, paediatric and pregnancy-related concerns, cone-beam computed tomography, radiation protection, myths and misconceptions, and questions involving imaging-related choices or professional consultation.

A structured expert content-review process informed by Lawshe's framework was undertaken (28). Seven volunteer Oral and Maxillofacial Radiologists independently reviewed each question for relevance and necessity. These experts participated only in the question-set review and were not involved in the subsequent scoring of AI-generated responses; they were neither study authors nor response evaluators.

Experts classified each question as “essential,” “useful but not essential,” or “not necessary.” Because the original item-level classifications and detailed Content Validity Ratio calculation records were not retained, item-level values are not reported and the final question set is described as expert-reviewed rather than as a formally validated instrument.

The structured review process was informed by Lawshe's approach and previous questionnaire-development studies (28–30).

The expert classifications were used together with qualitative feedback to guide item selection and refinement; no retrospective reconstruction of missing item-level classifications or CVR values was attempted.

Following expert review, the initial pool of 57 questions was refined for relevance, clarity and topic coverage. Duplicate questions and those addressing substantially overlapping concerns were removed or consolidated, while ambiguous wording was revised. Twenty questions were retained to cover common patient-facing concerns about radiation exposure and safety, the necessity and frequency of imaging, pregnancy, paediatric imaging, cone-beam computed tomography and radiation protection. Selection was based on qualitative expert feedback and coverage of these topics rather than a numerical Content Validity Ratio threshold. The final set is therefore described as expert-reviewed rather than as a formally validated questionnaire.

### 2.2 Question refinement and standardization

Following incorporation of the experts’ qualitative feedback, the question set was reviewed by the research team to ensure clarity, consistency, and readability before finalization. Minor editorial refinements were made where appropriate, without altering the intended meaning of the questions. The complete final expert-reviewed question set is provided in Supplementary File 1.

An item-specific evidence framework was developed for the 20 retained questions to support transparent and reproducible interpretation of the evaluation domains. For each question, it identifies the relevant authoritative sources, essential evidence-based content, acceptable contextual qualifications, potentially misleading or unsafe statements, and triage applicability. The complete framework is provided as Supplementary File 3.

Questions were reviewed to minimize technical terminology and improve comprehensibility for members of the general public by the research team (PV, MK). Particular attention was paid to maintaining language that reflected realistic patient concerns and information-seeking behaviour. The final question set was considered representative of common patient queries relating to dental radiology and radiation safety.

### 2.3 AI platform configurations and response generation

Three widely used consumer-facing AI platforms were selected based on their accessibility and increasing use for healthcare information seeking. The platform names and configurations displayed to the investigators were:

- Investigator-recorded, user-visible ChatGPT-5.5 configuration (OpenAI), with the reasoning (“Thinking”) mode enabled
- Investigator-recorded, user-visible Gemini 3 Flash free configuration (Google)
- Investigator-recorded, user-visible Microsoft Copilot 2026 free configuration (Microsoft)

We selected the most capable configuration available to the investigators within their existing access arrangements. An existing paid ChatGPT subscription provided access to the Thinking-enabled configuration, whereas Gemini and Copilot were accessed through their free tiers. This pragmatic selection did not produce computationally equivalent configurations. Consequently, observed differences may reflect access tier and reasoning settings as well as other platform characteristics.

ChatGPT, Gemini, and Microsoft Copilot are consumer-facing product interfaces and their displayed names do not necessarily identify fixed or independently verifiable underlying model versions. Accordingly, the platform names reported here refer to the interface labels and configurations visible to the investigators during data collection.

In this manuscript, “platform configuration” denotes the specific consumer-facing product interface, access tier, and settings evaluated. “LLM” refers generally to the underlying class of large language models; the exact proprietary backend implementation of a consumer platform may not be independently verifiable.

The tested configurations were not computationally equivalent because ChatGPT was accessed with the reasoning (“Thinking”) configuration enabled, whereas Gemini and Copilot were accessed through their available free configurations. Consequently, the observed differences cannot be attributed solely to the underlying model families and may also reflect differences in access tier, reasoning allocation, interface behaviour, retrieval functions, or undisclosed backend routing.

Data collection was conducted in late April 2026, following the public release of GPT-5.5 on 23 April 2026. To ensure standardisation, all interactions were conducted using the same device, browser, and internet environment. A new chat session was initiated for every question across all platforms. Chat history, memory functions, and prior conversational context were disabled to prevent carry-over effects and contextual bias.

Exact interaction dates and times for individual responses, contemporaneous screenshots, exported conversations, browser-build details, browsing status, historical account and interface metadata, backend model snapshots, and language or regional settings were not retained. These details could not be retrospectively verified and are therefore reported as unavailable rather than reconstructed.

Each question was entered independently using a standardized prompt:

“Please answer the following dental radiology question as you would explain it to a patient. Use clear, simple, and patient-friendly language.”

The same prompt was used across all AI platforms without modification. No follow-up questions, clarifications, corrections, or additional instructions were provided. Exactly one response was generated for each question from each platform and recorded verbatim, resulting in 60 responses (20 questions×3 platform configurations). Each AI-generated response was copied and pasted directly into an Excel workbook, with the question and corresponding platform identified. The final workbook contains all 60 response texts and forms the basis of Supplementary File 5. During revision, the supplementary response texts were checked against this workbook for consistency. Screenshots and exported conversations were not retained, which limits independent verification against the original platform conversations. No repeated generations were performed. Therefore, the study evaluated the specific outputs generated under the tested conditions and did not assess within-platform stochastic variability, repeatability, or response stability.

### 2.4 Coded evaluation of AI responses

AI-generated responses were evaluated independently by three postgraduate-qualified Oral and Maxillofacial Radiologists with a minimum of five years of combined clinical and academic experience. Before formal scoring, all evaluators received the same written rubric and detailed scoring instructions and reviewed the domain definitions and score descriptors to establish a shared interpretation of the assessment criteria. This calibration consisted of reviewing the scoring framework before formal evaluation. The rubric and scoring instructions are provided in Supplementary File 2.

Platform names were removed from the material supplied to the evaluators, and responses were labelled using A, B and C codes. The coding key was held exclusively by the corresponding author and was not shared with the evaluators or other authors during evaluation. Original response formatting, including headings and other stylistic features, was retained without standardisation. Although explicit platform labels were concealed, these features may have permitted platform recognition. No formal assessment of blinding success was performed.

### 2.5 Evaluation framework

Responses were independently evaluated across seven domains using one rating item per domain. Accuracy, relevance, clarity, completeness, safety and triage appropriateness were scored from 1 to 5, with higher scores indicating more favourable ratings. Misinformation was scored from 0 to 4, with lower scores indicating less misinformation. The detailed rubric is provided in Supplementary File 2.

1. Accuracy
2. Relevance
3. Clarity
4. Completeness
5. Safety
6. Triage appropriateness
7. Presence of misinformation

The seven-domain rubric was developed by the research team specifically for this study to assess the informational quality and potential clinical implications of patient-facing dental radiology advice. The domains were selected in accordance with the study objectives and informed by the clinical and radiation-protection considerations identified in the authoritative sources used during question development. The domain-specific score descriptors distinguished the degree to which each response met the criterion for a domain, including the clinical importance of omissions or missing warnings. Detailed operational anchors in Supplementary File 2 clarify distinctions between adjacent scores, including minor versus clinically important omissions and generally safe advice versus advice lacking an important warning. The rubric was study-specific and was not treated as an independently validated psychometric instrument.

Although the evaluated domains were conceptually related, each was operationalized to address a different feature of the response. Accuracy assessed whether information that was presented was factually correct when compared with the item-specific evidence framework. Completeness assessed whether essential information was omitted, irrespective of the correctness of the information that was included. Misinformation assessed the presence and clinical importance of affirmative incorrect, misleading, fabricated, or materially ambiguous statements; omissions alone were not classified as misinformation unless they created a misleading impression. Safety assessed the potential consequence of relying on the response, including false reassurance, inappropriate avoidance of indicated imaging, unnecessary alarm, or delayed professional care. A factual error affected safety only when it had a plausible harm-related consequence. Relevance assessed whether the response directly addressed the question, while clarity assessed whether the information was understandable to a layperson. Evaluators were instructed to apply these definitions separately while recognizing that a single response feature could affect more than one domain when it independently satisfied the criteria for each domain.

The clarity and patient-facing quality assessments were performed by expert radiologists. No patients or members of the intended public audience participated in rubric development or response evaluation. Accordingly, clarity scores represent expert judgments of apparent comprehensibility rather than directly measured patient understanding.

Triage appropriateness was considered applicable only to Question 20, which explicitly required guidance regarding professional consultation and decision-making following a dentist's recommendation for radiographic imaging. The remaining 19 questions primarily requested general information and were therefore classified as not applicable for triage assessment. Routine inclusion of a general disclaimer or recommendation to consult a professional was not considered evidence of appropriate triage. For Question 20, triage was evaluated according to whether the response recommended proportionate professional discussion, supported informed decision-making, avoided unnecessary urgency, and did not encourage inappropriate refusal or delay of clinically indicated imaging.

### 2.6 Statistical analysis

Statistical analyses were performed using JASP software (Version 0.19.3; JASP Team, Amsterdam, the Netherlands) (31). Descriptive statistics were calculated for all evaluated domains. Because the ratings were ordinal and showed restricted distributions, medians, interquartile ranges, frequencies, and percentages were emphasized; means and standard deviations, where retained, were interpreted as supplementary descriptive summaries only. Exact sign tests, binomial confidence intervals, and Holm adjustments were verified using Python 3.12 with SciPy 1.17.0.

Normality of score distributions was assessed using the Shapiro–Wilk test. As the majority of variables demonstrated non-normal distributions (*p* < 0.05), non-parametric statistical methods were employed for inferential analyses.

For each question, platform configuration, and evaluation domain, the scores assigned independently by the three evaluators were averaged to provide one summary score for the corresponding fixed AI-generated response. Because the three evaluators assessed the same response, their ratings were regarded as correlated assessments rather than independent experimental observations. Accordingly, the 20 questions were treated as the primary paired experimental units for inferential analysis. Individual evaluator-level ratings were retained for descriptive distributional summaries and inter-rater reliability assessment but were not treated as 60 independent paired observations.

Because each question was evaluated across all three platform configurations under matched conditions, question-level evaluator-averaged scores were compared using the Friedman test for related samples. Effect sizes were calculated using Kendall's coefficient of concordance (Kendall's W) and interpreted as small (0.1–0.3), moderate (0.3–0.5), or strong (>0.5). Holm adjustment was applied across the six domain-level Friedman tests.

Following a statistically significant omnibus test, pairwise platform comparisons were conducted at the question level using two-sided exact sign tests. For each comparison, the numbers of questions with higher, lower, or tied evaluator-averaged scores were determined; tied questions were excluded from the exact sign test. A sign-based dominance measure was calculated as D = (H−L)/(H + L), where H and L represent the numbers of discordant questions favouring the first- and second-listed platform configurations, respectively. Tied questions were excluded. Exact 95% confidence intervals were obtained by transforming the corresponding exact binomial proportion limits using D = 2p−1. Holm adjustment was applied across the three pairwise comparisons within each domain. For misinformation, the comparison direction was reversed so that positive D values indicate less misinformation. D is undefined when all questions are tied.

A cumulative-link mixed-effects model incorporating question- and evaluator-level clustering was considered. However, complete or quasi-complete separation and the highly restricted score distributions produced unstable or non-finite parameter estimates in several domains. The alternative analysis therefore treated the 20 questions as the primary paired experimental units, avoiding the assumption that the three evaluations of each fixed response constituted independent observations.

To examine potential overlap among the evaluation domains, Spearman rank correlations were calculated using the evaluator-averaged scores for the 60 AI-generated responses. Triage appropriateness was excluded because it was applicable to only one question. Correlations were treated as descriptive assessments of convergent or potentially redundant domains rather than as additional inferential tests.

Because triage appropriateness was applicable only to Question 20, it was excluded from inferential analysis and summarized descriptively.

Inter-rater reliability was assessed separately for each applicable domain using the 60 AI-generated responses as the units of analysis and the three radiologists as fixed evaluators. A two-way mixed-effects, absolute-agreement model was used to calculate both the single-measure ICC [ICC(A,1)] and average-measure ICC [ICC(A,3)]. The single-measure ICC estimates the reliability of a rating provided by one evaluator, whereas the average-measure ICC estimates the reliability of the mean of the three evaluator ratings. Ninety-five percent confidence intervals were obtained using response-level bootstrap resampling (32). ICC was retained because the analysis evaluated absolute agreement and the reliability of the three-evaluator mean used in the primary question-level analysis. Nevertheless, because ICC treats the ordered categories as approximately interval-scaled measurements, its application to five-point ordinal ratings is acknowledged as a methodological limitation. Triage appropriateness was excluded from ICC analysis because it was applicable to only three platform responses under Question 20, which was insufficient for a stable domain-level reliability estimate.

ICCs were calculated across responses pooled from all three platform configurations. Because much of the score variation in several domains occurred between configurations, these estimates primarily reflect agreement on differences between configurations rather than discrimination between responses within a configuration. They do not establish score validity or successful evaluator blinding.

To assess potential floor and ceiling effects, the complete distribution of individual evaluator ratings was examined for each platform–domain combination. The frequencies and percentages assigned to every possible score were calculated, including the percentages receiving the minimum and maximum scale scores. Question-level evaluator-averaged scores were also compared between platform configurations. For each pairwise comparison, the numbers of questions on which the first platform received a higher, lower, or tied score were reported. These question-level higher, lower, and tied counts formed the basis of the exact paired comparisons and also illustrated the extent of tied rankings and the discriminatory capacity of the scoring instrument.

During revision, a *post hoc* descriptive review of the 60 retained responses identified shielding statements inconsistent with the item-specific evidence framework and ADA/AAOMR recommendations (33, 34). Categories were defined for this *post hoc* review and counted once per response per category, allowing overlap: S1, outdated or unqualified current-use advice for named lead aprons or thyroid collars; S2, claims of complete or near-complete protection or elimination of radiation risk through shielding; S3, two-apron pregnancy advice without a clinical rationale; and S4, outdated shielding advice attributed to current ADA guidance. General references to unnamed protective measures and appropriately qualified historical or local-policy descriptions were excluded from S1. Flagged statements, guideline references and original evaluator scores are provided in Supplementary File 7. This review was separate from the original evaluation and did not alter the recorded ratings.

All statistical tests were two-tailed, and statistical significance was established at *p* < 0.05. The overall study design and methodological workflow are summarized in Figure 1.

**Figure 1:**
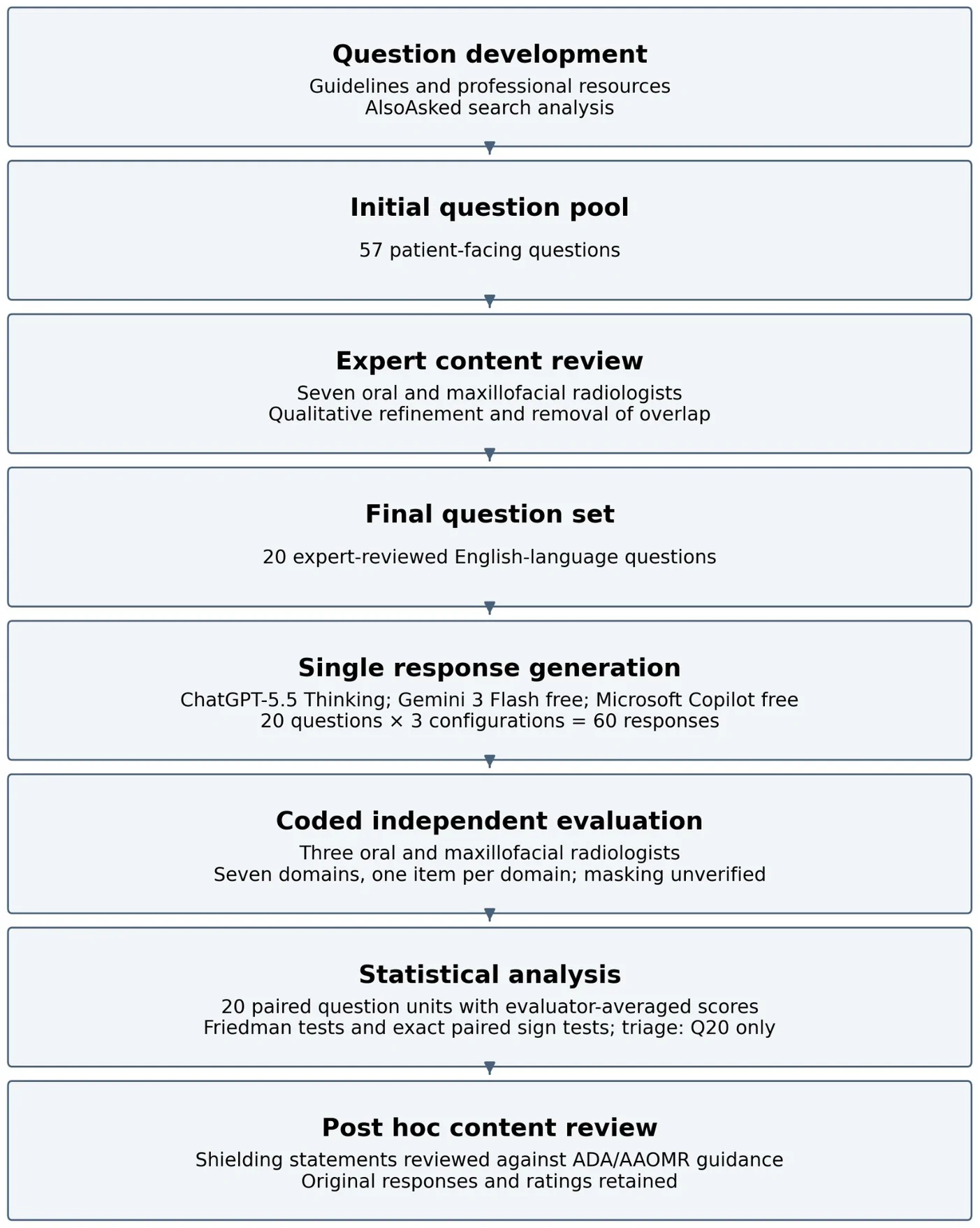
Study design and methodological workflow.

Reporting was guided by the applicable items of the TRIPOD-LLM reporting guideline for studies evaluating large language models in healthcare (35).

## 3 Results

A total of 60 fixed AI-generated responses were evaluated by three radiologists, producing 180 evaluator-level records. Six domains were analysed across 20 matched questions; triage was assessed descriptively for Question 20 only. Pooled single-measure ICCs ranged from moderate to excellent and were influenced by between-configuration score differences. They do not establish score validity, within-configuration discrimination or repeatability of AI outputs.

The final question set predominantly comprised short, general informational questions concerning radiation exposure and safety. Only Question 20 was classified as explicitly requiring consultation or decision-related guidance. Consequently, the findings primarily represent performance on common patient-facing informational questions and should not be extended to complex, conflicting, or case-specific clinical scenarios.

### 3.1 Inter-rater reliability

Inter-rater reliability was calculated separately for each domain using the 60 AI-generated responses as the units of analysis and the three radiologists as fixed evaluators (Table 1). Pooled single-measure ICCs ranged from moderate to excellent, with clarity showing moderate agreement (ICC = 0.696). These estimates describe agreement in the recorded ratings but are influenced by between-configuration score differences and should not be interpreted as evidence of reliable discrimination between individual responses within each configuration.

**Table 1:** Single- and average-measure inter-rater reliability across evaluation domains.

Domain	Response units	ICC(A,1), 95% CI	Interpretation	ICC(A,3), 95% CI	Interpretation
Accuracy	60	0.976 (0.923–1.000)	Excellent	0.992 (0.973–1.000)	Excellent
Relevance	60	0.850 (0.747–0.941)	Good	0.945 (0.899–0.979)	Excellent
Clarity	60	0.696 (0.508–0.821)	Moderate	0.873 (0.756–0.932)	Good
Completeness	60	0.961 (0.894–1.000)	Excellent	0.987 (0.962–1.000)	Excellent
Safety	60	0.958 (0.905–1.000)	Excellent	0.986 (0.966–1.000)	Excellent
Misinformation	60	0.881 (0.814–0.933)	Good	0.957 (0.929–0.977)	Excellent

ICC(A,1), two-way mixed-effects absolute-agreement single-measure ICC; ICC(A,3), two-way mixed-effects absolute-agreement average of three evaluators. Confidence intervals were obtained using response-level bootstrap resampling. Each analysis pooled the 60 responses comprising 20 questions and three platform configurations. Triage appropriateness was not included because it was applicable to only three responses under Question 20.

### 3.2 Comparative analysis of LLM performance

The distributions of individual ratings for each platform configuration across the evaluated domains are presented in Table 2. The selected ChatGPT configuration had the greatest concentration of favourable ratings for accuracy, relevance, and safety; Gemini generally showed intermediate distributions, while Copilot had lower distributions in several domains.

**Table 2:** Distribution of individual ratings across platform configurations.

Domain	ChatGPT (*n* = 60 ratings)	Gemini (*n* = 60 ratings)	Copilot (*n* = 60 ratings)
Accuracy	Score 5: 60 ratings (100%)	Score 3: 1 rating (1.7%); Score 4: 59 ratings (98.3%)	Score 4: 60 ratings (100%)
Relevance	Score 4: 3 ratings (5.0%); Score 5: 57 ratings (95.0%)	Score 3: 1 rating (1.7%); Score 4: 48 ratings (80.0%); Score 5: 11 ratings (18.3%)	Score 3: 1 rating (1.7%); Score 4: 58 ratings (96.7%); Score 5: 1 rating (1.7%)
Clarity	Score 4: 35 ratings (58.3%); Score 5: 25 ratings (41.7%)	Score 4: 45 ratings (75.0%); Score 5: 15 ratings (25.0%)	Score 3: 18 ratings (30.0%); Score 4: 42 ratings (70.0%)
Completeness	Score 4: 60 ratings (100%)	Score 3: 2 ratings (3.3%); Score 4: 52 ratings (86.7%); Score 5: 6 ratings (10.0%)	Score 3: 59 ratings (98.3%); Score 4: 1 rating (1.7%)
Safety	Score 5: 60 ratings (100%)	Score 3: 2 ratings (3.3%); Score 4: 58 ratings (96.7%)	Score 3: 2 ratings (3.3%); Score 4: 58 ratings (96.7%)
Misinformation^a^	Score 0: 60 ratings (100%)	Score 0: 1 rating (1.7%); Score 1: 31 ratings (51.7%); Score 2: 28 ratings (46.7%)	Score 1: 41 ratings (68.3%); Score 2: 19 ratings (31.7%)

n, number of individual ratings.

Each platform received 60 individual ratings per domain: 20 responses evaluated independently by three evaluators. Score categories not displayed in the table received no ratings. For accuracy, relevance, clarity, completeness, and safety, higher scores indicate better performance. For misinformation, lower scores indicate better performance.

a Lower misinformation scores indicate better performance.

Question-level Friedman tests identified statistically significant differences among the three platform configurations across all evaluated domains (Table 3). These *p*-values indicate evidence of differences within this sample; their practical importance should be interpreted together with effect sizes, rating distributions, ceiling effects, and the numbers of tied question-level comparisons.

**Table 3:** Friedman test results comparing platform configurations (20 matched questions per domain).

Domain	*χ* ^2^	Holm Adjusted *p*-value	Kendall's W	Matched questions, n
Accuracy	39.38	<0.001	0.984	20
Relevance	29.66	<0.001	0.741	20
Clarity	17.80	<0.001	0.445	20
Completeness	36.81	<0.001	0.920	20
Safety	40.00	<0.001	1.000	20
Misinformation	30.40	<0.001	0.760	20

### 3.3 Accuracy

A significant question-level difference was observed in accuracy scores among the three platform configurations (χ²(2) = 39.38, Holm-adjusted *p* < 0.001, Kendall's W = 0.984). ChatGPT received more favourable ratings than Gemini and Copilot for all 20 questions (both D = 1.000, 95% CI 0.663 to 1.000; both Holm-adjusted *p* < 0.001). Gemini and Copilot were tied on 19 questions and differed on only one; this comparison was not significant (D = −1.000, 95% CI −1.000 to 0.950; Holm-adjusted *p* = 1.000).

### 3.4 Relevance

A significant question-level difference was observed in relevance scores (χ²(2) = 29.66, Holm-adjusted *p* < 0.001, Kendall's W = 0.741). ChatGPT received more favourable ratings than Gemini on 18 questions, less favourable ratings on one, and tied on one (D = 0.895, 95% CI 0.479 to 0.997; Holm-adjusted *p* < 0.001). ChatGPT also received more favourable ratings than Copilot on 19 questions and less favourable ratings on one (D = 0.900, 95% CI 0.503 to 0.997; Holm-adjusted *p* < 0.001). Gemini was more favourable than Copilot on five questions and tied on 15, but the comparison was not significant after Holm adjustment (D = 1.000, 95% CI −0.044 to 1.000; *p* = 0.063).

### 3.5 Clarity

Clarity scores differed significantly at the question level (χ²(2) = 17.80, Holm-adjusted *p* < 0.001, Kendall's W = 0.445). ChatGPT and Gemini did not differ significantly (10/7/3 questions higher/lower/tied; D = 0.176, 95% CI −0.342 to 0.631; Holm-adjusted *p* = 0.629). ChatGPT received more favourable ratings than Copilot (16/0/4; D = 1.000, 95% CI 0.588 to 1.000; Holm-adjusted *p* < 0.001), and Gemini received more favourable ratings than Copilot (10/0/10; D = 1.000, 95% CI 0.383 to 1.000; Holm-adjusted *p* = 0.004).

### 3.6 Completeness

A significant question-level difference was observed for completeness (χ²(2) = 36.81, Holm-adjusted *p* < 0.001, Kendall's W = 0.920). ChatGPT and Gemini did not differ significantly (1/2/17 questions higher/lower/tied; D = −0.333, 95% CI −0.983 to 0.811; Holm-adjusted *p* = 1.000). ChatGPT received more favourable ratings than Copilot for all 20 questions (D = 1.000, 95% CI 0.663 to 1.000; Holm-adjusted *p* < 0.001). Gemini received more favourable ratings than Copilot on 19 questions and tied on one (D = 1.000, 95% CI 0.647 to 1.000; Holm-adjusted *p* < 0.001).

### 3.7 Safety

Safety demonstrated the strongest question-level omnibus effect (χ²(2) = 40.00, Holm-adjusted *p* < 0.001, Kendall's W = 1.000). ChatGPT received more favourable ratings than both Gemini and Copilot for all 20 questions (both D = 1.000, 95% CI 0.663 to 1.000; both Holm-adjusted *p* < 0.001). Gemini and Copilot were tied for all 20 questions; consequently, their sign-based dominance measure was not estimable and no difference was detected (Holm-adjusted *p* = 1.000).

### 3.8 Triage appropriateness

Individual evaluator ratings and descriptive summaries for triage appropriateness are presented in Table 4. Triage appropriateness was assessed only for Question 20, which explicitly required guidance regarding professional consultation and decision-making. ChatGPT received ratings of 4 from all three evaluators. Gemini 3 Flash received ratings of 4, 4, and 3, while Microsoft Copilot received ratings of 3, 4, and 4. All three platform configurations therefore had a median score of 4. Because only one question was considered triage-applicable, these results were summarized descriptively, and no inferential comparison was performed. These findings do not establish broader differences in the triage capabilities of the evaluated platforms.

**Table 4:** Descriptive triage-appropriateness ratings for question 20.

Platform configuration	Evaluator 1	Evaluator 2	Evaluator 3	Median (range)
ChatGPT	4	4	4	4 (4–4)
Google Gemini	4	4	3	4 (3–4)
Microsoft Copilot	3	4	4	4 (3–4)

Triage appropriateness was applicable only to Question 20: “If my dentist recommends an x-ray but I am worried about radiation, what should I do?” Ratings are reported descriptively because only one question was triage-applicable. No inferential comparison was performed.

### 3.9 Misinformation

Misinformation scores differed significantly at the question level (χ²(2) = 30.40, Holm-adjusted *p* < 0.001, Kendall's W = 0.760). After reversing the comparison direction so that positive values represented less misinformation, ChatGPT was more favourable than Gemini and Copilot for all 20 questions (both D = 1.000, 95% CI 0.663 to 1.000; both Holm-adjusted *p* < 0.001). Gemini was more favourable than Copilot on eight questions and less favourable on 12 (D = −0.200, 95% CI −0.618 to 0.279; Holm-adjusted *p* = 0.503).

The primary question-level exact paired analysis supported the omnibus findings while avoiding treatment of the three correlated evaluator ratings as independent observations (Table 5). ChatGPT received more favourable ratings than both Gemini and Copilot for accuracy, relevance, safety, and misinformation, and more favourable ratings than Copilot for clarity and completeness. Gemini received more favourable ratings than Copilot for clarity and completeness. No significant differences were identified between ChatGPT and Gemini for clarity or completeness, or between Gemini and Copilot for accuracy, relevance, safety, or misinformation. Several comparisons contained many tied questions, so effect estimates were interpreted together with the higher, lower, and tied counts.

**Table 5:** Question-level exact paired comparisons across platform configurations.

Domain	Comparison	Questions: higher/lower/tied	Sign-based dominance D (95% CI)	Holm-adjusted *p*-value
Accuracy	ChatGPT vs. Gemini	20/0/0	1.000 (0.663 to 1.000)	<0.001
	ChatGPT vs. Copilot	20/0/0	1.000 (0.663 to 1.000)	<0.001
	Gemini vs. Copilot	0/1/19	−1.000 (−1.000 to 0.950)	1.000
Relevance	ChatGPT vs. Gemini	18/1/1	0.895 (0.479 to 0.997)	<0.001
	ChatGPT vs. Copilot	19/1/0	0.900 (0.503 to 0.997)	<0.001
	Gemini vs. Copilot	5/0/15	1.000 (−0.044 to 1.000)	0.063
Clarity	ChatGPT vs. Gemini	10/7/3	0.176 (−0.342 to 0.631)	0.629
	ChatGPT vs. Copilot	16/0/4	1.000 (0.588 to 1.000)	<0.001
	Gemini vs. Copilot	10/0/10	1.000 (0.383 to 1.000)	0.004
Completeness	ChatGPT vs. Gemini	1/2/17	−0.333 (−0.983 to 0.811)	1.000
	ChatGPT vs. Copilot	20/0/0	1.000 (0.663 to 1.000)	<0.001
	Gemini vs. Copilot	19/0/1	1.000 (0.647 to 1.000)	<0.001
Safety	ChatGPT vs. Gemini	20/0/0	1.000 (0.663 to 1.000)	<0.001
	ChatGPT vs. Copilot	20/0/0	1.000 (0.663 to 1.000)	<0.001
	Gemini vs. Copilot	0/0/20	Not estimable	1.000
Misinformation^a^	ChatGPT vs. Gemini	20/0/0	1.000 (0.663 to 1.000)	<0.001
	ChatGPT vs. Copilot	20/0/0	1.000 (0.663 to 1.000)	<0.001
	Gemini vs. Copilot	8/12/0	−0.200 (−0.618 to 0.279)	0.503

Comparisons used evaluator-averaged scores for the 20 matched questions, which were the primary paired experimental units. Higher/lower/tied indicates the numbers of questions for which the first-listed platform received a more favourable, less favourable, or equal score, respectively. Tied questions were excluded from the two-sided exact sign test. The sign-based dominance measure (D) was calculated from discordant question-level pairs as (H-L)/(H + L), and exact 95% confidence intervals were derived from the corresponding binomial proportions. *P*-values were adjusted using the Holm procedure across the three pairwise platform comparisons within each domain.

a For misinformation, lower original scores indicated better performance; the comparison direction was reversed so that higher and a positive D consistently represented less misinformation.

Misinformation was scored from 0 to 4, with lower scores indicating less misinformation. Representative statements included Gemini Q11, which suggested using “two lead aprons” during pregnancy, and Copilot Q10, which stated that lead aprons and thyroid collars are “always used.” These recommendations are inconsistent with contemporary guidance against routine patient shielding in dental radiography (33, 34). For Gemini Q11, Evaluators 2 and 3 assigned misinformation scores of 2 alongside accuracy and safety scores of 4; for Copilot Q10, all three evaluators assigned this combination. Overall, all 28 Gemini and 19 Copilot ratings with misinformation score 2 also received accuracy and safety scores of 4. Although the domains assessed different aspects of each response, these combinations are not fully consistent with the accuracy rubric, which reserves score 4 for minor imprecision. The original ratings were retained without retrospective alteration.

#### 3.9.1 *Post hoc* shielding-content review

Outdated or unqualified advice for named shielding occurred in 1/20 ChatGPT responses (5%), 12/20 Gemini responses (60%) and 11/20 Copilot responses (55%). Complete or near-complete protection or risk-elimination claims occurred in 6/20 Gemini responses (30%) and 1/20 Copilot responses (5%), with none identified in ChatGPT responses under this criterion. Gemini Q11 suggested two lead aprons (1/20, 5%), and Gemini Q17 attributed outdated shielding advice to ADA guidance (1/20, 5%). Categories overlap. Gemini Q19 was included because it recommends thyroid-collar use and claims near-zero thyroid exposure. Full question-level findings are provided in Supplementary File 7.

### 3.10 Score distributions and ceiling effects

Examination of the complete individual-rating distributions demonstrated substantial ceiling effects and restricted score variation in several domains (Table 2). All 60 ChatGPT accuracy and safety ratings were 5, representing a 100% ceiling rate in both domains. ChatGPT also received the maximum relevance score for 57 of 60 ratings (95.0%). For misinformation, all 60 ChatGPT ratings were 0, indicating that the original evaluators recorded no misinformation and a 100% concentration at the most favourable end of the reverse-scored scale. Ceiling effects were less pronounced for clarity, for which maximum ratings were recorded for 41.7% of ChatGPT and 25.0% of Gemini ratings. These scores do not establish that the response texts contained no outdated or misleading statements.

Question-level comparisons further demonstrated substantial numbers of tied scores, particularly for completeness and in comparisons between Gemini and Copilot (Table 5). ChatGPT received a higher evaluator-averaged accuracy and safety score than both comparator platforms for all 20 questions. However, ChatGPT and Gemini had tied completeness scores for 17 of 20 questions. Gemini and Copilot were tied for safety on all 20 questions and for accuracy on 19 of 20 questions. These findings indicate that the very large Kendall's W values partly reflect uniform rank ordering within this limited and relatively homogeneous question set and should not be interpreted as evidence of general superiority beyond the evaluated outputs.

### 3.11 Overall performance

For the single outputs evaluated under the specified testing conditions, the selected ChatGPT configuration received the most favourable ratings in several domains, particularly accuracy, relevance, and safety. Gemini showed intermediate ratings, and Copilot had lower ratings in several domains. Figure 2 shows the distributions of the original individual evaluator ratings. Each six-domain panel contains correlated ratings of 20 responses by three evaluators per configuration; triage is shown separately for Question 20. These distributions describe recorded ratings and do not establish score validity or general platform superiority.

**Figure 2:**
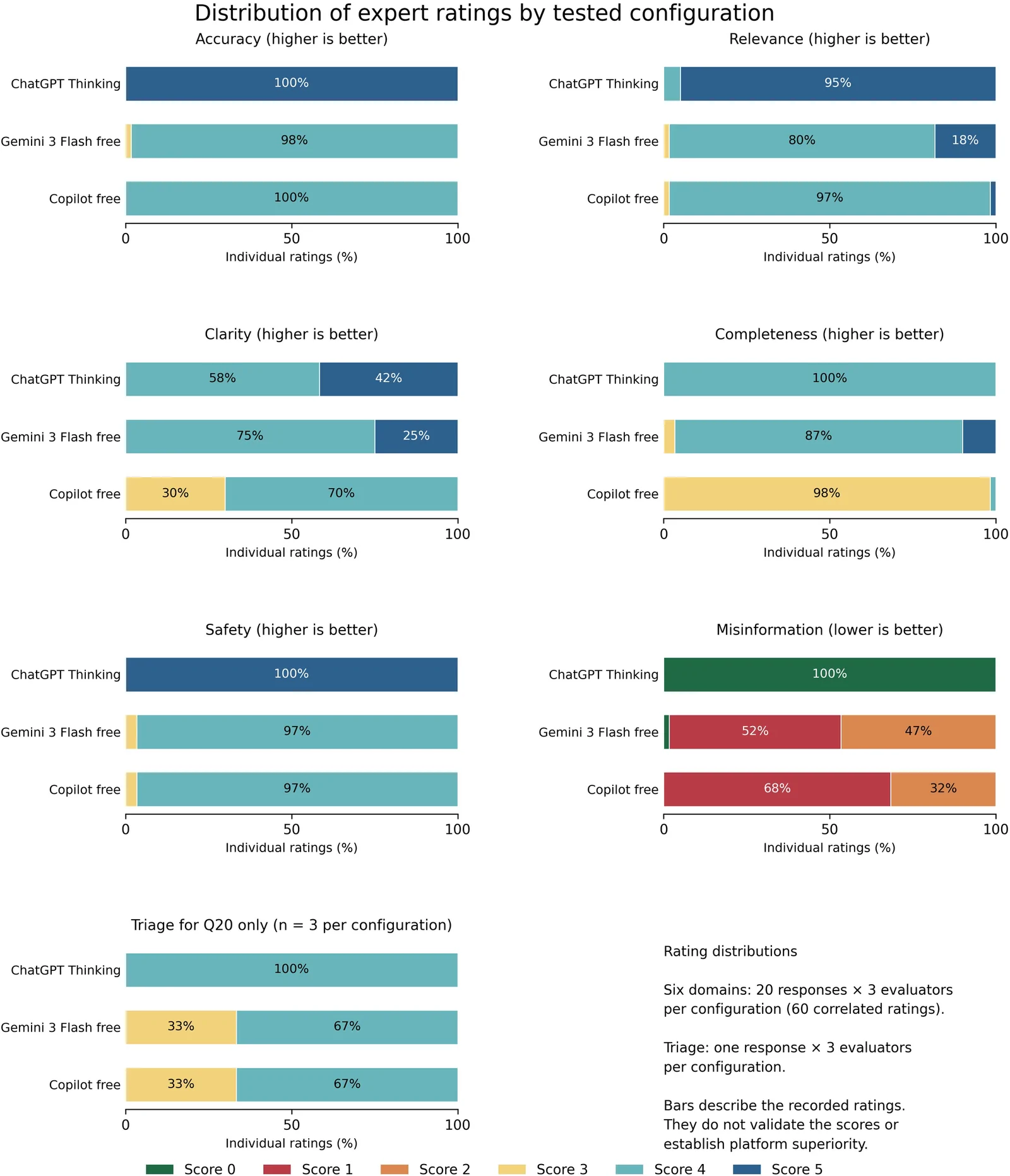
Distributions of individual evaluator ratings by tested configuration. Accuracy, relevance, clarity, completeness and safety use scores 1–5 (higher is more favourable); misinformation uses scores 0–4 (lower is more favourable). Each six-domain panel contains 60 correlated ratings per configuration (20 responses×three evaluators). Triage is displayed separately for Question 20, with three ratings per configuration. The chart is descriptive and does not depict independent observations, score validity or general platform superiority.

### 3.12 Inter-domain correlations

Spearman correlations demonstrated varying degrees of association among the evaluated domains. Accuracy was strongly correlated with safety (*ρ* = 0.985) and relevance (*ρ* = 0.859), while safety was also strongly correlated with relevance (*ρ* = 0.876). Because lower misinformation scores indicated better performance, misinformation was negatively correlated with accuracy (*ρ* = −0.809) and safety (*ρ* = −0.784). Clarity demonstrated a moderate correlation with completeness (*ρ* = 0.633). Other correlations ranged from weak-to-moderate to strong. These findings indicate that several domains captured related aspects of response quality and should not be interpreted as statistically independent outcomes (Table 6).

**Table 6:** Spearman correlations among evaluator-averaged domain scores across 60 AI responses.

Domain	Accuracy	Relevance	Clarity	Completeness	Safety	Misinformation
Accuracy	1.000	0.859	0.465	0.454	0.985	−0.809
Relevance	0.859	1.000	0.418	0.489	0.876	−0.703
Clarity	0.465	0.418	1.000	0.633	0.462	−0.440
Completeness	0.454	0.489	0.633	1.000	0.455	−0.309
Safety	0.985	0.876	0.462	0.455	1.000	−0.784
Misinformation	−0.809	−0.703	−0.440	−0.309	−0.784	1.000

Correlations were calculated using evaluator-averaged scores for the 60 responses. Lower misinformation scores represent better performance. Triage appropriateness was excluded because it was applicable to only one question.

## 4 Discussion

This study compared expert ratings of single patient-facing responses generated by three selected consumer-facing AI platform configurations for 20 dental radiology questions. Differences were observed across several evaluated domains under the specific testing conditions. However, because the tested configurations differed in access tier, reasoning allocation, and potentially other interface or backend characteristics, the findings should not be interpreted as demonstrating the intrinsic or general superiority of one underlying model family over another.

These findings extend previous evaluations of AI-generated oral-radiology patient education (12) and the comparison of the same three platforms for paediatric dental advice by overlapping authors (13). The additional contribution lies in the dental radiation-safety focus, explicit assessment of safety, item-specific evidence framework and question-level paired analysis. However, the small informational question set and single-response design limit the breadth of this contribution.

Under the specific testing conditions, the selected ChatGPT configuration received higher ratings in several domains. The findings concern single outputs from unequal consumer platform configurations and therefore do not demonstrate general model superiority. Access tier, reasoning settings, interface behaviour, retrieval functions, and undisclosed backend routing may all have contributed. Previous studies likewise report task- and platform-dependent performance across dental applications (11–13, 36–41).

Accuracy is essential for patient-facing health information. Under the study conditions, the selected ChatGPT responses received higher accuracy ratings than the Gemini and Copilot responses. Errors concerning radiation risk, imaging frequency, or diagnostic necessity could plausibly contribute to unnecessary concern or inappropriate decisions, although patient behaviour and clinical outcomes were not measured. Related variability in AI-generated dental advice has been reported previously (9, 10, 37–40).

Differences were also observed in relevance and completeness. The selected ChatGPT responses more consistently addressed the questions directly, whereas some comparator responses were less focused or omitted expected information. Similar variation in contextual relevance and patient-oriented communication has been reported in dental evaluations of LLMs (36, 40). These qualities may be especially important for people with limited health literacy (16, 17), but this study did not directly assess comprehension among patients or other intended users.

ChatGPT and Gemini showed comparable results for clarity and completeness in the primary question-level pairwise analyses. This suggests that the evaluated outputs from both configurations often presented information in an apparently understandable form, consistent with reports that several contemporary LLMs can produce readable patient-education content (12, 36). Because radiologists rather than patients judged clarity, these findings should not be interpreted as evidence of actual patient comprehension.

Triage appropriateness was examined only for Question 20, which asked how a concerned patient should respond to a dentist's recommendation for radiographic imaging. The remaining questions primarily requested general information and were not suitable for triage assessment. The triage results were therefore descriptive and cannot establish broader platform-level triage performance. A routine recommendation to consult a professional was not automatically considered appropriate; advice also had to be proportionate and avoid unnecessary urgency, inappropriate refusal, or delay.

Although misinformation ratings differed among the platform configurations, inaccurate or misleading patient-facing health information remains a concern. Previous investigations have documented fabricated references, inconsistent factual reliability, and variable performance on complex clinical topics (12, 38–40). In dental radiology, incorrect statements about radiation exposure, pregnancy, paediatric imaging, cancer risk, or the need for radiographs could plausibly influence perceptions and decisions. The observed differences cannot be attributed to a specific model property because training, alignment, access tier, reasoning settings, retrieval, and backend routing were not independently evaluated.

A response may contain a broadly accurate central message alongside a specific misleading statement, and factual inaccuracy does not necessarily imply an equivalent degree of potential harm. Nevertheless, this distinction does not fully reconcile clinically relevant misinformation with an accuracy score reserved for minor imprecision. The observed score combinations therefore indicate limitations in the application of the rubric, and favourable aggregate ratings should not be interpreted as demonstrating the absence of clinically relevant content errors.

Contemporary ADA/AAOMR recommendations advise against routine abdominal and thyroid shielding in dental radiography, including for children and pregnant patients, while applicable local regulatory requirements must be considered (33, 34). Statements presenting shields as mandatory or guaranteeing complete protection can reinforce outdated expectations. Patients may become concerned or reluctant to accept otherwise justified imaging when a clinic appropriately omits shielding. These are plausible implications, not patient outcomes measured in this study. Clinicians should explain clinical justification, dose optimisation and current local requirements. The flagged ChatGPT Q17 advice also shows that favourable aggregate ratings do not establish the absence of outdated content.

The pooled ICCs require cautious interpretation. Restricted within-configuration score variation and substantial between-configuration differences can produce high agreement estimates even when ratings discriminate poorly between individual responses within a configuration. These estimates do not establish successful masking or construct validity and do not resolve concerns about halo effects or the validity of the scores.

The inter-domain correlation analysis demonstrated substantial overlap among several outcomes. In particular, accuracy and safety were almost perfectly correlated, while misinformation was strongly inversely correlated with both domains. This is clinically understandable because inaccurate or misleading information can also create safety concerns. Nevertheless, the constructs were not considered entirely interchangeable: accuracy concerned factual correctness, misinformation concerned affirmative misleading content, and safety concerned the potential consequence of relying on the advice. Similarly, the moderate correlation between clarity and completeness suggests that understandable responses often contained more of the expected information, although these constructs remained conceptually distinct. Accordingly, the domain-level results should be interpreted as related perspectives on response quality rather than as seven independent sources of evidence.

The primary inferential analysis treated the 20 questions as paired experimental units, while individual evaluator ratings were used descriptively and for agreement assessment. Question-level exact comparisons provided ordinal effect estimates and 95% confidence intervals without treating the three ratings of each fixed response as independent observations. Nevertheless, several comparisons contained substantial numbers of tied questions, reflecting the restricted five-point scale and pronounced ceiling effects. Complete or quasi-complete separation prevented stable estimation using conventional cumulative-link mixed-effects models. The estimates should therefore be interpreted together with the reported higher, lower, and tied question counts and the limited sample of 20 questions.

From a broader perspective, the findings illustrate both the potential usefulness and limitations of conversational AI for access to oral-health information, particularly where access to dental professionals may be limited (14, 15). Recent studies suggest that AI systems may serve as educational adjuncts for patients and dental students when used with appropriate professional oversight (13, 40). However, the variability observed among the evaluated outputs indicates that the selected platform configurations did not provide uniformly comparable expert-rated informational quality and safety. AI-generated information should therefore be regarded as a supplementary educational resource rather than a substitute for professional dental assessment and guidance.

### 4.1 Clinical implications

The findings suggest that selected consumer-facing AI platform configurations can generate apparently understandable information about common dental radiology questions. However, clarity and usefulness were evaluated by radiologists rather than patients. The study did not measure whether patients understood, trusted, accepted, or acted upon the information, nor whether the responses reduced anxiety or influenced acceptance of radiographic imaging. AI-generated information should therefore be regarded as a potential supplementary educational resource requiring professional oversight rather than as a demonstrated method of improving patient understanding or influencing patient behaviour.

## 5 Limitations

This study evaluated one response per platform for each of 20 predominantly short, English-language informational questions using a single prompt and no follow-up interaction. The question set may not represent the breadth or complexity of real clinical enquiries, and single generations cannot establish within-platform repeatability or response stability. Only three consumer-facing platform configurations were tested, and they were not computationally equivalent: ChatGPT used a reasoning-enabled configuration, whereas Gemini and Copilot were accessed through available free configurations. Differences therefore cannot be attributed solely to underlying model families.

Additional limitations concern measurement and generalizability. Ratings showed marked ceiling effects, restricted variation, and many ties, which limited discrimination and can yield extreme sign-based dominance estimates based on few discordant pairs. The study-specific rubric was not independently validated or formally piloted; pooled inter-rater agreement is influenced by between-configuration differences and does not establish within-configuration discrimination or construct validity. Triage appropriateness applied to only one question. Radiologists, rather than patients, judged clarity, and the study measured neither patient comprehension nor behavioural or clinical outcomes. Response-specific evaluator explanations were not recorded, preventing retrospective determination of the reasons for inconsistencies between misinformation and accuracy scores. No evaluator reassessment was undertaken, and the original ratings were preserved. Correlated domains should not be regarded as independent confirmations of performance. Finally, incomplete preservation of contemporaneous interface metadata, exact timestamps, and exported conversations limits exact replication because consumer platforms and backend systems change over time. These limitations restrict the conclusions to the specific outputs, configurations, question set, and testing period examined. The inferential analysis was based on only 20 matched questions. Averaging the three correlated evaluator ratings provided a response-level summary but did not increase the number of independent experimental units. Retained formatting and stylistic features may have enabled platform recognition despite removal of platform names and withholding of the coding key; possible unblinding and halo effects cannot be excluded. The *post hoc* content review was not a blinded independent evaluator reassessment or a validated error-detection procedure. No objective readability assessment was performed.

### 5.1 Future directions

Future studies should include repeated generations, equivalent access tiers and reasoning settings, verified evaluator masking, more complex questions, response-specific error adjudication and direct assessment by patients. These measures would help establish response stability, validity of ratings and patient comprehension.

## 6 Conclusion

Under the specific testing conditions, expert ratings differed among single responses generated by the three selected consumer-facing AI platform configurations for 20 English-language patient-facing dental radiology questions. The selected ChatGPT configuration received higher ratings in several domains, although substantial ceiling effects, tied ratings, overlapping outcomes, unverified masking, score–content inconsistencies, unequal platform configurations, and the absence of repeated generations limit interpretation. These findings describe only the evaluated outputs and do not establish general platform superiority, stable performance, patient comprehension, clinical benefit, or suitability as a replacement for professional dental advice. Future studies should include repeated generations, equivalent platform configurations, more complex clinical scenarios, and direct evaluation by patients and other intended users.

## Data Availability

The complete AI-generated responses are provided in Supplementary File 5. Individual evaluator ratings, the coding key, data dictionary and analysis-ready dataset are provided in Supplementary File 6.

## Supplementary material

Supplementary File 1 Final expert-reviewed patient-facing dental radiology question set.

Supplementary File 2 Detailed scoring rubric and operational anchors.

Supplementary File 3 Item-specific evidence framework for the 20 dental radiology questions.

Supplementary File 4 TRIPOD-LLM applicability checklist.

Supplementary File 5 Complete question set and 60 AI-generated responses.

Supplementary File 6 Individual evaluator ratings, coding key, data dictionary, and analysis-ready dataset.

Supplementary File 7 Post hoc descriptive review of shielding-related statements in the 60 retained responses.
