# Supplementary material for: Expert-rated accuracy and safety of patient-facing dental radiology responses from three AI platform configurations: a comparative evaluation
Source: Front Oral Health. 2026 Oct 21;7:1937905. doi: 10.3389/froh.2026.1937905 (PMC13733446; doi:10.3389/froh.2026.1937905)
Supplement: Supplementary file 1 [file Supplementaryfile1.docx]

Supplementary File 1 Final expert reviewed question set

**Section A: General Safety & Understanding**

1. Are dental X-rays safe for routine check-ups?
2. Do dental X-rays expose me to a lot of radiation?
3. Can dental X-rays increase my risk of cancer?
4. Are digital dental X-rays safer than older ones?

**Section B: Frequency & Necessity**

5. How often should dental X-rays be taken?
6. Do I need X-rays if I don’t have any symptoms?
7. What happens if I refuse dental X-rays?
8. Are repeated dental X-rays harmful over time?

**Section C: Pregnancy & Children**

9. Are dental X-rays safe during pregnancy?
10. Can dental X-rays harm my unborn baby?
11. Should I delay dental treatment if I need X-rays during pregnancy?
12. Are dental X-rays safe for children?

**Section D: Advanced Imaging & Risk Communication**

13. What is the difference between a dental X-ray and CBCT?
14. Is CBCT more dangerous than regular dental X-rays?
15. When is CBCT really necessary?

**Section E: Protection & Safety Measures**

16. Why is a lead apron used during dental X-rays?
17. Is a thyroid collar necessary?

**Section F: Myths & Misconceptions**

18. Is it safer to avoid dental X-rays completely?
19. Do dental X-rays cause long-term health problems?

**Section G: Clinical Decision / Triage**

20. If my dentist recommends an X-ray but I am worried about radiation, what should I do?
