# Supplementary material for: Expert-rated accuracy and safety of patient-facing dental radiology responses from three AI platform configurations: a comparative evaluation
Source: Front Oral Health. 2026 Oct 21;7:1937905. doi: 10.3389/froh.2026.1937905 (PMC13733446; doi:10.3389/froh.2026.1937905)
Supplement: Supplementary file 4 [file Supplementaryfile4.docx]

Supplementary File 4

**TRIPOD-LLM Applicability Checklist**

*Associated manuscript: Expert-Rated Accuracy and Safety of Single Patient-Facing Dental Radiology Responses from Selected Consumer AI Platform Configurations: A Comparative Study*

# Applicability statement

This checklist records the TRIPOD-LLM items applicable to this external evaluation of consumer-facing large language model outputs. Items concerning model development, fine-tuning, proprietary training data, or prediction-model deployment are marked not applicable. Manuscript section names are provided because final page numbers may change during production.

| **Item** | **Reporting topic** | **Study-specific completion** | **Manuscript location** | **Status** |
| --- | --- | --- | --- | --- |
| 1 | Title | Identifies an expert-rated evaluation of single patient-facing responses generated by selected consumer platform configurations. | Title | Reported |
| 2 | Abstract | States the design, tested configurations, single-generation procedure, evaluation methods, principal findings, and limitations. | Abstract | Reported |
| 3a | Healthcare context and rationale | Explains the dental-radiology information context and rationale for evaluating patient-facing responses. | Introduction | Reported |
| 3b | Target population and intended use | Identifies members of the public seeking dental-radiology information and does not claim clinical deployment. | Introduction | Reported |
| 4 | Objectives | Specifies comparative expert evaluation of fixed outputs; no model development or clinical validation is claimed. | End of Introduction | Reported |
| 5a-b | Evaluation data | Describes the source and development of the 20 English-language questions, three configurations, and 60 generated responses. | Methods: Question Set; Response Generation | Reported |
| 5c | Relevant dates | Reports the question-search date and late-April-2026 response period; unavailable exact interaction timestamps are acknowledged. | Methods: Response Generation | Partially reported; limitation stated |
| 5d-e | Preprocessing, quality checks and missingness | Responses were recorded verbatim without follow-up or post-processing; the handling of applicable outcomes is described. | Methods | Reported |
| 6a | Platform/model identification | Reports the displayed platform/configuration labels and explains that proprietary backend identities could not be independently verified. | Methods: AI Platform Configurations | Partially reported; limitation stated |
| 6b | Development, training and alignment | The evaluated platforms were externally developed proprietary systems; development and training details were not available to the investigators. | Not applicable to this external evaluation | Not applicable |
| 7 | Prompting strategy | Provides the exact standardized prompt and independent-session procedure. | Methods: Response Generation | Reported |
| 8 | Human oversight and evaluation | Describes evaluator qualifications, coded response assessment, withholding of platform names and the coding key, the written rubric, evidence framework, and calibration procedure. | Methods: Expert evaluation; Supplementary Files 2, 3 and 6 | Reported |
| 9 | Reference standard and outcomes | Defines the evaluation domains and supplies the detailed rubric and item-specific evidence framework. | Methods; Supplementary Files 2 and 3 | Reported |
| 10 | Sample size | Reports 20 questions multiplied by three configurations, producing 60 responses rated by three evaluators. | Methods and Results | Reported |
| 11 | Statistical analysis | Describes matched ordinal analyses, effect estimates and confidence intervals, multiplicity control, sensitivity analyses, correlations, score distributions, and reliability analyses. | Methods: Statistical Analysis | Reported |
| 12 | Ethics and governance | Reports institutional ethics approval and describes coded evaluator assessment and data handling. | Methods | Reported |
| 13 | Open science and availability | The complete AI-generated response dataset is provided in Supplementary File 5. All 180 individual evaluator rating records, the coding key, data dictionary, and 60-row analysis-ready dataset are provided in Supplementary File 6. | Data availability statement; Supplementary Files 5 and 6 | Reported |
| 14 | Study flow and analysed data | Reports the number of generated responses and the analysis units used for each analysis. | Results | Reported |
| 15 | Performance results | Reports domain-specific results, effect estimates, confidence intervals, adjusted p-values, rating distributions, and tied comparisons. | Results; Tables 2-6 | Reported |
| 16 | Error and robustness analyses | Reports misinformation findings, domain overlap, the post hoc descriptive shielding review, and sensitivity analyses based on individual evaluator ratings. | Methods and Results; Supplementary File 7 | Reported |
| 17 | Interpretation | Restricts interpretation to the evaluated outputs and configurations and distinguishes statistical findings from practical significance. | Discussion | Reported |
| 18 | Limitations and generalizability | Addresses single generation, unequal configurations, unavailable historical metadata, ceiling effects, domain overlap, question scope, and absence of patient validation. | Limitations | Reported |
| 19 | Implications and future research | Recommends repeated generations, equivalent configurations, complex scenarios, and direct evaluation involving intended users. | Clinical Implications; Future Directions | Reported |

# Reference

Gallifant J, Afshar M, Ameen S, et al. The TRIPOD-LLM reporting guideline for studies using large language models. Nature Medicine. 2025;31(1):60-69. doi:10.1038/s41591-024-03425-5.

Guideline source: https://doi.org/10.1038/s41591-024-03425-5
